# Adversarial Robustness with Partial Isometry

**DOI:** 10.3390/e26020103

**Published:** 2024-01-24

**Authors:** Loïc Shi-Garrier, Nidhal Carla Bouaynaya, Daniel Delahaye

**Affiliations:** 1 ENAC, Université de Toulouse, 31400 Toulouse, France; delahaye@recherche.enac.fr; 2 Department of Electrical and Computer Engineering, Rowan University, Glassboro, NJ 08028, USA; bouaynaya@rowan.edu

**Keywords:** adversarial robustness, information geometry, fisher information metric, multi-class classification

## Abstract

Despite their remarkable performance, deep learning models still lack robustness guarantees, particularly in the presence of adversarial examples. This significant vulnerability raises concerns about their trustworthiness and hinders their deployment in critical domains that require certified levels of robustness. In this paper, we introduce an information geometric framework to establish precise robustness criteria for $l_{2}$ white-box attacks in a multi-class classification setting. We endow the output space with the Fisher information metric and derive criteria on the input–output Jacobian to ensure robustness. We show that model robustness can be achieved by constraining the model to be partially isometric around the training points. We evaluate our approach using MNIST and CIFAR-10 datasets against adversarial attacks, revealing its substantial improvements over defensive distillation and Jacobian regularization for medium-sized perturbations and its superior robustness performance to adversarial training for large perturbations, all while maintaining the desired accuracy.

## 1. Introduction

One of the primary motivations for investigating machine learning robustness stems from the susceptibility of neural networks to adversarial attacks, wherein small perturbations in the input data can deceive the network into making the wrong decision. These adversarial attacks have been shown to be both ubiquitous and transferable [1,2,3]. Beyond posing a security threat, adversarial attacks underscore the glaring lack of robustness in machine learning models [4,5]. This deficiency in robustness is a critical challenge as it undermines trustworthiness in machine learning systems [6].

In this paper, we shed an information geometric perspective to adversarial robustness in machine learning models. We show that robustness can be achieved by encouraging the model to be isometric in the orthogonal space of the kernel of the pullback Fisher information metric (FIM). We subsequently formulate a regularization defense method for adversarial robustness. While our focus is on $l_{2}$ white-box attacks within multi-class classification tasks, the method’s applicability extends to more general settings, including unrestricted attacks and black-box attacks across various supervised learning tasks. The regularized model is evaluated on MNIST and CIFAR-10 datasets against projected gradient descent (PGD) $l_{\infty }$ attacks and AutoAttack [7] with $l_{\infty }$ and $l_{2}$ norms. Comparisons with the unregularized model, defensive distillation [8], Jacobian regularization [9], and Fisher information regularization [10] show significant improvement in robustness. Moreover, the regularized model is able to ensure robustness against larger perturbations compared to adversarial training.

The remainder of this paper is organized as follows. Section 2 introduces notations, notions of adversarial machine learning, and definitions related to geometry. Then, we derive a sufficient condition for adversarial robustness at a given sample point. Section 3 presents our method for approximating the robustness condition, which involves promoting model isometry in the orthogonal complement of the kernel of the pullback of the FIM. In Section 4, several experiments are presented to evaluate the proposed method. Section 5 discusses the results in the context of related work on adversarial defense. Finally, Section 6 concludes the paper and outlines potential extensions of this research. Appendix A provides the proof of the results stated in the main text.

## 2. Notations and Definitions

### 2.1. Notations

Let $d,c\in N^{*}$ such that d≥c>1. Let m=c−1. In the learning framework, *d* will be the dimension of the input space, while *c* will be the number of classes. The range of a matrix *M* is denoted as rg(M). The rank of *M* is denoted as rk(M). The Euclidean norm (i.e., $l_{2}$ norm) is denoted as ∥·∥. We use the notation $\delta_{ij}=1$ if i=j and 0 otherwise. We denote the components of a vector *v* by $v^{i}\in R$ with a superscript. Smooth means $C^{\infty }$.

### 2.2. Adversarial Machine Learning

An adversarial attack is any strategy aiming at deliberately altering the expected behavior of a model or extracting information from a model. In this work, we focus on attacks performed at inference time (i.e., after training), sometimes referred to as evasion attacks. The most well-known evasion attacks are gradient-based. Such gradient-based attacks all follow the same idea that we explain thereafter.

To reach good accuracy and generalization, a machine learning model *f* (with input *x* and parameter *w*) is typically trained by minimizing a loss function L(y,f(x,w)) with respect to the parameters *w* of the model. In its simpler form, the loss function quantifies the error between the prediction of the model f(x,w) and ground-truth *y*. Given a clean input $x_{0}$, an adversarial example $x_{*}$ can be crafted by maximizing the loss function L(y,f(x,w)), starting from $x_{0}$ and using gradient ascent $x_{t+1}-x_{t}\propto \nabla_{x}L\left(y,f\left(x,w\right)\right)$, where the gradient is computed with respect to the input *x* (and not the parameter *w* as during training). In order for $x_{*}$ to be an adversarial example, $x_{0}$ and $x_{*}$ must be close to each other according to some dissimilarity measure, typically a $l_{p}$ norm. An adversarial example $x_{*}$ is successful if the model *f* classifies $x_{*}$ differently from $x_{0}$. Some well-known gradient-based attacks include the fast gradient sign method [2] and projected gradient descent [3].

Adversarial attacks can be classified according to their threat model. White-box attacks assume that the adversary has perfect knowledge of the targeted model, including access to the training data, model architecture, and model parameters. Such an adversary can directly compute the gradient $\nabla_{x}L\left(y,f\left(x,w\right)\right)$ of the targeted model and craft adversarial examples. More realistic threat models are classified as gray-box or black-box attacks, where some or all of the information is unknown to the adversary. In this work, we use both white-box attacks as well as simple gray-box attacks where the adversary can access the training data and model architecture, but not the model parameters. To craft such gray-box adversarial examples, another model is trained with the same data and architecture. Then, white-box attacks are performed on this model. Finally, the adversarial examples can be transferred to the targeted model.

Adversarial robustness aims to build models that classify both $x_{*}$ and $x_{0}$ with the same class while preserving sufficient accuracy for the clean examples $x_{0}$. Various defenses have been proposed to improve adversarial robustness. The most efficient defense is called adversarial training, which was first described in [2] and further developed in [3]. The idea behind adversarial training is to obtain the parameters $w_{*}$ of the trained model as:

$$w_{*}=\underset{w}{\mathrm{arg min}}\max_{\epsilon \in \Delta (x)}L\left(y,f\left(x+\epsilon ,w\right)\right),$$

in place of the original parameters ${\mathrm{arg min}}_{w}L\left(y,f\left(x,w\right)\right)$. The set Δ(x) is a set of allowed adversarial attacks for *x*, e.g., a $l_{2}$ ball with a given radius (or budget). In practice, adversarial training is performed by adding adversarial examples to the training set, thus providing a lower bound for $\max_{\epsilon \in \Delta (x)}L\left(y,f\left(x+\epsilon ,w\right)\right)$.

### 2.3. Geometrical Definitions

Consider a multi-class classification task. Let $X\subseteq R^{d}$ be the input domain, and let Y={1,…,c}⊂N be the set of labels for the classification task. For example, in MNIST, we have $X={\left[0,1\right]}^{d}$ (with d=784) and c=10. We assume that X is a *d*-dimensional embedded smooth connected submanifold of $R^{d}$. Let m=c−1.

**Definition** **1** (Probability simplex). *Define the probability simplex of dimension m by*

(1) $$\Delta^{m}=\left\{\theta \in R^{m}:\forall k\in \left\{1,\ldots ,m\right\},\theta^{k}>0\;\mathrm{and}\;\sum_{i=1}^{m}\theta^{i}<1\right\}.$$

*$\Delta^{m}$ is a smooth submanifold of $R^{c}$ of dimension m. We can see $\theta =(\theta^{1},\ldots ,\theta^{m})$ as a coordinate system from $\Delta^{m}$ to $R^{m}$. Then, let us define $\theta^{c}=1-\sum_{i=1}^{m}\theta^{i}$.*

A machine learning model (e.g., a neural network) is often seen as assigning a label y∈Y to a given input x∈X. Instead, in this work, we see a model as assigning the parameters of a random variable *Y* to a given input x∈X. The random variable *Y* has a probability density function $p_{\theta }$ belonging to the family of *c*-dimensional categorical distributions $S=\{p_{\theta }:\theta \in \Delta^{m}\}$.

S can be endowed with a differentiable structure by using $p_{\theta }\in S\mapsto \left(\theta^{1},\ldots ,\theta^{m}\right)\in R^{m}$ as a global coordinate system. Hence, S becomes a smooth manifold of dimension *m* (more details on this construction can be found in [11], Chapter 2). We can identify $p_{\theta }$ with $\left(\theta^{1},\ldots ,\theta^{m}\right)$.

We see any machine learning model as a smooth map $f:X\to \Delta^{m}$ that assigns to an input x∈X, the parameters $\theta =f\left(x\right)\in \Delta^{m}$ of a *c*-dimensional categorical distribution $p_{\theta }\in S$. In practice, a neural network produces a vector of logits s(x). Then, these logits are transformed into the parameters θ with the softmax function: θ=softmax(s(x)).

In order to study the sensitivity of the predicted $f\left(x\right)\in \Delta^{m}$ with respect to the input x∈X, we need to be able to measure distances both in X and in $\Delta^{m}$. In order to measure distances on smooth manifolds, we need to equip each manifold with a Riemannian metric.

First, we consider $\Delta^{m}$. As described above, we see $\Delta^{m}$ as the family of categorical distributions. A natural Riemannian metric for $\Delta^{m}$ (i.e., a metric that reflects the statistical properties of $\Delta^{m}$) is the Fisher information metric (FIM).

**Definition** **2** (Fisher information metric). *For each $\theta \in \Delta^{m}$, the Fisher information metric (FIM) g defines a symmetric positive-definite bilinear form $g_{\theta }$ over the tangent space $T_{\theta }\Delta^{m}$. In the standard coordinates of $R^{c}$, for all $\theta \in \Delta^{m}$ and all tangent vectors $v,w\in T_{\theta }\Delta^{m}$, we have*

(2) $$g_{\theta }\left(v,w\right)=v^{T}G_{\theta }w,$$

*where $G_{\theta }$ is the Fisher information matrix for parameter $\theta \in \Delta^{m}$, defined by*

(3) $$G_{\theta ,ij}=\frac{\delta_{ij}}{\theta^{i}}+\frac{1}{\theta^{c}}.$$

*For any $\theta \in \Delta^{m}$, the matrix $G_{\theta }$ is symmetric positive-definite and non-singular (see Proposition 1.6.2 in [12]). The FIM induces a distance on $\Delta^{m}$, called the Fisher–Rao distance, denoted as $d(\theta_{1},\theta_{2})$ for any $\theta_{1},\theta_{2}\in \Delta^{m}$.*

The FIM has two remarkable properties. First, it is the “infinitesimal distance” of the relative entropy, which is the loss function used to train a multi-class classification model. More precisely, if *D* is the relative entropy (also known as the Kullback–Leibler divergence) and if *d* is the Fisher–Rao distance, then given two distributions $\theta_{1}$ and $\theta_{2}$, we have (see Theorem 4.4.5 in [12]):

$$D\left(\theta_{1}\left|\right|\theta_{2}\right)=\frac{1}{2}d^{2}\left(\theta_{1},\theta_{2}\right)+o\left(d^{2}\left(\theta_{1},\theta_{2}\right)\right).$$

The same result can be restated infinitesimally using the FIM *g*, as follows:

(4) $$D\left(\theta ||\theta +d\theta \right)=\frac{1}{2}g_{\theta }\left(d\theta ,d\theta \right)+o\left(g_{\theta }\left(d\theta ,d\theta \right)\right),$$

where dθ is seen as a tangent vector of $T_{\theta }S$.

The other remarkable property of the FIM is Chentsov’s theorem [13], claiming that the FIM is the unique Riemannian metric on $\Delta^{m}$, which is invariant under sufficient statistics (up to a multiplicative constant). Informally, the FIM is the only Riemannian metric that is statistically meaningful. In [14], Amari and Nagaoka state a more general result. Along with the FIM, they introduce a family of affine connections parameterized by a real parameter α, called the α-connections. Theorem 2.6 in [14] states that an affine connection is invariant under sufficient statistics if and only if it is an α-connection for some α∈R. In other words, the α-connections are the only affine connections that have a statistical meaning. While Equation (4) gives the second-order approximation of the relative entropy, an α-connection can be seen as the third-order term in the Taylor approximation of some divergence [14]. More precisely, a given α-connection can be canonically associated with a unique divergence (while the second-order term is always given by the FIM). If α=±1, the canonical divergences are the relative entropy and its dual (obtained by switching the arguments in $D(\theta_{2}||\theta_{1})$). More generally, for α≠0, the canonical divergence is not symmetric. The only canonical divergence that is symmetric is obtained for α=0, and is precisely the square of the Fisher–Rao distance. Thus, the Fisher–Rao distance is the only statistically meaningful distance. This provides a motivation for using the Fisher–Rao distance to measure lengths in $\Delta^{m}$.

Now, we consider X. Since we are studying adversarial robustness, we need a metric that formalizes the idea that two close data points must be “indistinguishable” from a human perspective (or any other relevant perspective). A natural choice is the Euclidean metric induced from $R^{d}$ on X.

**Definition** **3** (Euclidean metric). *We consider the Euclidean space $R^{d}$ endowed with the Euclidean metric $\bar{g}$. It is defined in the standard coordinates of $R^{d}$ for all $x\in R^{d}$ and for all tangent vectors $v,w\in T_{x}R^{d}$ by*

(5) $${\bar{g}}_{x}\left(v,w\right)=v^{T}w,$$

*thus, its matrix is the identity matrix of dimension d, denoted as $I_{d}$. The Euclidean metric induces a distance on $R^{d}$ that we will denote with the $l_{2}$-norm: $∥x_{1}-x_{2}∥$ for any $x_{1},x_{2}\in R^{d}$.*

From now on, we fix:

- A smooth map $f:\left(X,\bar{g}\right)\to \left(\Delta^{m},g\right)$. We denote by $f^{i}$ the *i*-th component of *f* in the standard coordinates of $R^{c}$.
- A point x∈X.
- A positive real number ϵ>0.

Define the Euclidean open ball centered at *x* with radius ϵ by

(6) $$\bar{b}\left(x,\epsilon \right)=\left\{z\in R^{d}:∥z-x∥<\epsilon \right\}.$$

**Figure 1:**
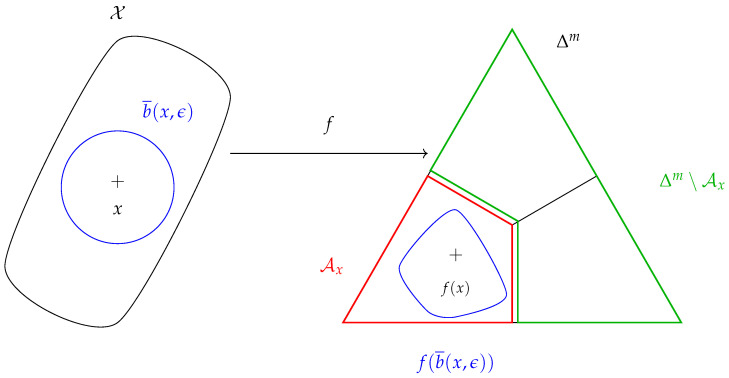
ϵ-robustness at *x* is enforced if and only if $f\left(\bar{b}\left(x,\epsilon \right)\right)\subseteq A_{x}$.

**Definition** **4.** *Define the set (Figure 1):*

(7) $$A_{x}=\left\{\theta \in \Delta^{m}:\arg\max_{i}\theta^{i}=\arg\max_{i}f^{i}\left(x\right)\right\}.$$

*For simplicity, assume that f(x) is not on the “boundary” of $A_{x}$, such that $\arg\max_{i}f^{i}\left(x\right)$ is well-defined.*

The set $A_{x}$ is the subset of distributions of $\Delta^{m}$ that have the same class as f(x).

**Figure 2:**
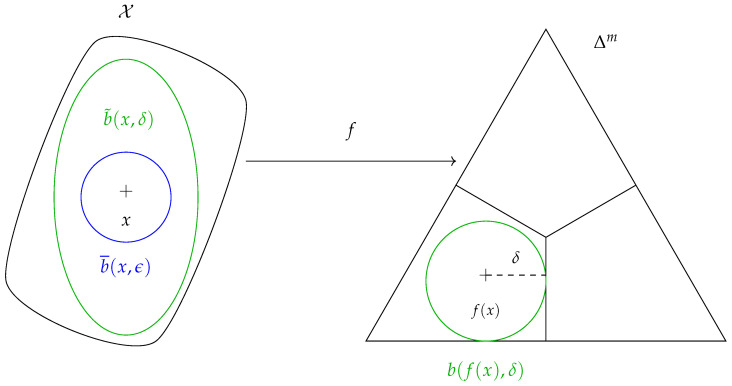
ϵ-robustness at *x* is enforced if $\bar{b}\left(x,\epsilon \right)\subseteq \tilde{b}\left(x,\delta \right)$.

**Definition** **5** (Geodesic ball of the FIM). *Let δ>0 be the Fisher–Rao distance between f(x) and $\Delta^{m}\setminus A_{x}$ (Figure 2), i.e., the Fisher–Rao distance between f(x) and the closest distribution of $\Delta^{m}$ with a different class.*

*Define the geodesic ball centered at $f\left(x\right)\in \Delta^{m}$ with radius δ by*

(8) $$b\left(f\left(x\right),\delta \right)=\left\{\theta \in \Delta^{m}:d\left(f\left(x\right),\theta \right)\leq \delta \right\}.$$

*In Section 3.3, we propose an efficient approximation of δ.*

**Definition** **6** (Pullback metric). *On X, define the pullback metric $\tilde{g}$ of g by f. In the standard coordinates of $R^{d}$, $\tilde{g}$ is defined for all tangent vectors $v,w\in T_{x}X$ by*

(9) $${\tilde{g}}_{x}\left(v,w\right)=v^{T}J_{x}^{T}G_{f(x)}J_{x}w,$$

*where $J_{x}$ is the Jacobian matrix of f at x (in the standard coordinates of $R^{d}$ and $R^{c}$). Define the matrix of ${\tilde{g}}_{x}$ in the standard coordinates of $R^{d}$ by*

(10) $${\tilde{G}}_{x}=J_{x}^{T}G_{f(x)}J_{x}.$$

**Definition** **7** (Geodesic ball of the pullback metric). *Let $\tilde{d}$ be the distance induced by the pullback metric $\tilde{g}$ on $R^{d}$. We can define the geodesic ball centered at x with radius δ by*

(11) $$\tilde{b}\left(x,\delta \right)=\left\{z\in R^{d}:\tilde{d}\left(x,z\right)\leq \delta \right\}.$$

*Note that the radius δ is the Fisher–Rao distance between f(x) and $\Delta^{m}\setminus A_{x}$ as defined in Definition 5.*

### 2.4. Robustness Condition

**Definition** **8** (Robustness). *We say that f is ϵ-robust at x if*

(12) $$\forall z\in R^{d},∥z-x∥<\epsilon \Rightarrow f\left(z\right)\in A_{x}.$$

*Equivalently, we can write (Figure 1):*

(13) $$f\left(\bar{b}\left(x,\epsilon \right)\right)\subseteq A_{x}.$$

**Proposition** **1** (Sufficient condition for robustness). *If $\bar{b}\left(x,\epsilon \right)\subseteq \tilde{b}\left(x,\delta \right)$, then f is ϵ-robust at x (Figure 2).*

Our goal is to start from Proposition 1 and make several assumptions in order to derive a condition that can be efficiently implemented.

Working with geodesic balls $\bar{b}\left(x,\epsilon \right)$ and $\tilde{b}\left(x,\delta \right)$ is intractable, so our first assumption consists of using an “infinitesimal” condition by restating Proposition 1 in the tangent space $T_{x}X$ instead of working directly on X. In $T_{x}X$, define the Euclidean ball of radius ϵ by

(14) $${\bar{B}}_{x}\left(0,\epsilon \right)=\left\{v\in T_{x}X:{\bar{g}}_{x}\left(v,v\right)=v^{T}v\leq \epsilon^{2}\right\}.$$

Similarly, in $T_{x}X$, define the ${\tilde{g}}_{x}$-ball of radius δ by

(15) $${\tilde{B}}_{x}\left(0,\delta \right)=\left\{v\in T_{x}X:{\tilde{g}}_{x}\left(v,v\right)=v^{T}{\tilde{G}}_{x}v\leq \delta^{2}\right\}.$$

**Assumption** **1.** *We replace Proposition 1 by*

(16) $${\bar{B}}_{x}\left(0,\epsilon \right)\subseteq {\tilde{B}}_{x}\left(0,\delta \right).$$

**Proposition** **2.** *Equation (16) is equivalent to*

(17) $$\forall v\in T_{x}X,\;{\tilde{g}}_{x}\left(v,v\right)\leq \frac{\delta^{2}}{\epsilon^{2}}{\bar{g}}_{x}\left(v,v\right).$$

Since m<d, the Jacobian matrix $J_{x}$ has a rank smaller or equal to *m*. Thus, since $G_{f(x)}$ has full rank, ${\tilde{G}}_{x}=J_{x}^{T}G_{f(x)}J_{x}$ has a rank of at most *m* (when $J_{x}$ has a rank of *m*).

**Assumption** **2.** *The Jacobian matrix $J_{x}$ has a full rank equal to m.*

Using Assumptions 1 and 2, the constant rank theorem ensures that for small enough δ, *f* is ϵ-robust at *x*. However, contrary to Proposition 1, Assumption 1 does not offer any guarantee on the ϵ-robustness at *x* for arbitrary δ.

## 3. Derivation of the Regularization Method

In this section, we derive a condition for robustness (Proposition 4), which can be implemented as a regularization method. Then, we provide two useful results for the practical implementation of this method: an explicit formula for the decomposition of the FIM as $G=P^{T}P$ (Section 3.2), and an easy-to-compute upper-bound of δ, i.e., the Fisher–Rao distance between f(x) and $\Delta^{m}\setminus A_{x}$ (Section 3.3).

### 3.1. The Partial Isometry Condition

In order to simplify the notations, we replace

- $J_{x}$ with *J*, which is a full-rank m×d real matrix.
- $G_{f(x)}$ with *G*, which is an m×m symmetric positive definite real matrix.
- ${\tilde{G}}_{x}$ with $\tilde{G}$, which is a d×d symmetric positive-semidefinite real matrix.

We define $D={\left(\ker\left(\tilde{G}\right)\right)}^{⊥}$. We will use the two following facts.

**Fact** **1.**
(18) $$D=\mathrm{rg}\left(J^{T}\right)={\left(\ker(J)\right)}^{⊥}={\left(\ker(J^{T}GJ)\right)}^{⊥}$$

**Fact** **2.** *$J^{T}GJ$ is symmetric positive semidefinite. Thus, by the spectral theorem, the eigenvectors associated with its nonzero eigenvalues are all in $D=\mathrm{rg}(J^{T})$.*

*In particular, since rk(J)=m, there exists an orthonormal basis of $T_{x}X$, denoted as $B=(e_{1},\ldots ,$$e_{m},e_{m+1},\ldots ,e_{d})$, such that each $e_{i}$ is an eigenvector of $J^{T}GJ$, and such that $\left(e_{1},\ldots ,e_{m}\right)$ is a basis of $D=\mathrm{rg}(J^{T})$ and $\left(e_{m+1},\ldots ,e_{d}\right)$ is a basis of ker(J).*

The set $D=\mathrm{rg}(J^{T})$ is an *m*-dimensional subspace of $T_{x}X$. ${\tilde{g}}_{x}$ does not define an inner product on $T_{x}X$ because $\tilde{G}$ has a nontrivial kernel of dimension d−m. In particular, the set ${\tilde{B}}_{x}\left(0,\delta \right)$ is not bounded, i.e., it is a cylinder rather than a ball. However, when restricted to *D*, ${\tilde{g}}_{x}{|}_{D}$ defines an inner product. We define the restriction of ${\tilde{B}}_{x}\left(0,\delta \right)$ to *D*:

(19) $${\tilde{B}}_{D}\left(0,\delta \right)=\left\{v\in D:v^{T}\tilde{G}v\leq \delta \right\},$$

and similarly, we define the restriction of ${\bar{B}}_{x}\left(0,\epsilon \right)$ to *D*:

(20) $${\bar{B}}_{D}\left(0,\epsilon \right)=\left\{v\in D:v^{T}v\leq \epsilon^{2}\right\}.$$

Assume that *f* is such that Equation (16) holds (i.e., ${\bar{B}}_{x}\left(0,\epsilon \right)\subseteq {\tilde{B}}_{x}\left(0,\delta \right)$). Moreover, assume that we are in the limit case defined as follows: for any perturbation size, we can find a smaller perturbation of *f* such that Equation (16) does not hold anymore. This limit case is equivalent to having ${\bar{B}}_{D}\left(0,\epsilon \right)={\tilde{B}}_{D}\left(0,\delta \right)$. In this case, ${\tilde{B}}_{x}\left(0,\delta \right)$ is the smallest possible ${\tilde{g}}_{x}$-ball (for the inclusion) such that Equation (16) holds. We noticed experimentally that enforcing this stronger criteria yields a larger robustifying effect. Thus, we make the following assumption:

**Assumption** **3.** *We replace Equation (16) with*

(21) $${\bar{B}}_{D}\left(0,\epsilon \right)={\tilde{B}}_{D}\left(0,\delta \right).$$

**Proposition** **3.** *Equation (21) is equivalent to*

(22) $$\forall v\in D,\;{\tilde{g}}_{x}\left(v,v\right)=\frac{\delta^{2}}{\epsilon^{2}}{\bar{g}}_{x}\left(v,v\right).$$

We can rewrite Equation (22) in matrix form:

(23) $$\forall v\in D,\;v^{T}\tilde{G}v=\frac{\delta^{2}}{\epsilon^{2}}v^{T}v.$$

In Section 3.2, we show how to exploit the properties of the FIM to derive a closed-form expression for a matrix $P\in {\mathrm{GL}}_{m}\left(R\right)$, such that $G=P^{T}P$. For now, we assume that we can easily access such a *P* and we are looking for a condition on *P* and *J*, which is equivalent to Equation (23).

**Proposition** **4.** *The following statements are equivalent:*

$$\begin{array}{cc} \left(i\right) & \;\forall u\in D,\;u^{T}J^{T}GJu=\frac{\delta^{2}}{\epsilon^{2}}u^{T}u,\\ \left(ii\right) & \;PJJ^{T}P^{T}=\frac{\delta^{2}}{\epsilon^{2}}I_{m}, \end{array}$$

*where $I_{m}$ is the identity matrix of dimension m×m.*

Proposition 4 constrains the matrix PJ to be a semi-orthogonal matrix (multiplied by a homothety matrix). A smooth map *f* between Riemannian manifolds $\left(X,\bar{g}\right)$ and $\left(\Delta^{m},g\right)$ is said to be (locally) isometric if the pullback metric (denoted $f^{*}g$) coincides with $\bar{g}$, i.e., $f^{*}g=\bar{g}$. Such a map *f* locally preserves distances. In our case, $f^{*}g=\tilde{g}$ is not a metric (since its kernel is non-trivial); thus, *f* cannot be an isometry. However, Equation (22) ensures that *f* locally preserves distances along directions spanned by *D*. Hence, *f* becomes a partial isometry, at least in the neighborhood of the training points.

Under the Assumptions 1–3, Equation (ii) in Proposition 4 implies robustness as defined in Definition 8. In other words, Equation (ii) is a sufficient condition for robustness. However, there is no reason for a neural network to satisfy Equation (ii). This is why we define the following regularization term:

(24) $$\alpha \left(x,\epsilon ,f\right)=\frac{1}{m^{2}}|||PJJ^{T}P^{T}-\frac{\delta^{2}}{\epsilon^{2}}I_{m}|||,$$

where |||·||| is any matrix norm, such as the Frobenius norm or the spectral norm. We use the Frobenius norm in the experiments of Section 4. To compute α(x,ϵ,f), we only need to compute the Jacobian matrix *J*, which can be efficiently achieved with backpropagation. Finally, the loss function is:

(25) $$L\left(y,x,\epsilon ,f\right)=l\left(y,f(x)\right)+\lambda \alpha \left(x,\epsilon ,f\right),$$

where *l* is the cross-entropy loss, and λ>0 is a hyperparameter controlling the strength of the regularization with respect to the cross-entropy loss. The regularization term α(x,ϵ,f) is minimized during training, such that the model is pushed to satisfy the sufficient condition of robustness.

### 3.2. Coordinate Change

In this subsection, we show how to compute the matrix *P* that was introduced in Proposition 4. To this end, we isometrically embed $\Delta^{m}$ into the Euclidean space $R^{c}$ using the following inclusion map:

$$\begin{array}{cc} \mu :\Delta^{m} & ⟶R^{c}\\ \left(\theta^{1},\ldots ,\theta^{m}\right) & \mapsto 2\left(\sqrt{\theta^{1}},\ldots ,\sqrt{\theta^{m}},\sqrt{1-\sum_{i=1}^{m}\theta^{i}}\right) \end{array}$$

We can easily see that μ is an embedding. If $S^{m}\left(2\right)$ is the sphere of radius 2 centered at the origin in $R^{c}$, then $\mu \left(\Delta^{m}\right)$ is the subset of $S^{m}\left(2\right)$, where all coordinates are strictly positive (using the standard coordinates of $R^{c}$).

**Proposition** **5.** *Let g be the Fisher information metric on $\Delta^{m}$ (Definition 2), and $\bar{g}$ be the Euclidean metric on $R^{c}$. Then μ is an isometric embedding of $\left(\Delta^{m},g\right)$ into $\left(R^{c},\bar{g}\right)$.*

Now, we use the stereographic projection to embed $\Delta^{m}$ into $R^{m}$:

$$\begin{array}{cc} \tau :\mu (\Delta^{m}) & ⟶R^{m}\\ \left(\mu^{1},\ldots ,\mu^{m},\mu^{c}\right) & \mapsto 2\left(\frac{\mu^{1}}{2-\mu^{c}},\ldots ,\frac{\mu^{m}}{2-\mu^{c}}\right), \end{array}$$

with $\mu^{c}=2\sqrt{1-\sum_{i=1}^{m}\theta^{i}}$.

**Proposition** **6.** *In the coordinates τ, the FIM is:*

(26) $$G_{\tau ,ij}=\frac{4}{{\left({1+∥\tau /2∥}^{2}\right)}^{2}}\delta_{ij}.$$

Let $\tilde{J}$ be the Jacobian matrix of $\tau \circ \mu :\Delta^{m}\to R^{m}$ at f(x). Then, we have:

(27) $$G={\tilde{J}}^{T}G_{\tau }\tilde{J}=\frac{4}{{\left({1+∥\tau /2∥}^{2}\right)}^{2}}{\tilde{J}}^{T}\tilde{J}.$$

Thus, we can choose:

(28) $$P=\frac{2}{{1+∥\tau /2∥}^{2}}\tilde{J}.$$

Write $f\left(x\right)=\theta =\left(\theta^{1},\ldots ,\theta^{m}\right)$ and $\theta^{c}=1-\sum_{i=1}^{m}\theta^{i}$. For simplicity, write $\tau^{i}\left(\theta \right)=\tau^{i}\left(\mu \left(\theta \right)\right)=2\sqrt{\theta^{i}}/\left(1-\sqrt{\theta^{c}}\right)$ for i=1,…,m. More explicitly, we have:

**Proposition** **7.** *For i,j=1,…,m:*

(29) $$P_{ij}=\frac{\delta_{ij}}{\sqrt{\theta^{i}}}-\frac{\tau^{i}\left(\theta \right)}{2\sqrt{\theta^{c}}}.$$

### 3.3. The Fisher–Rao Distance

In this subsection, we derive a simple upper-bound for δ (i.e., the Fisher–Rao distance between f(x) and $\Delta^{m}\setminus A_{x}$). In Proposition 5, we show that the probability simplex $\Delta^{m}$ endowed with the FIM can be isometrically embedded into the *m*-sphere of radius 2. Thus, the angle β between two distributions of coordinates $\theta_{1}$ and $\theta_{2}$ in $\Delta^{m}$ with $\mu_{1}=\mu \left(\theta_{1}\right)$ and $\mu_{2}=\mu \left(\theta_{2}\right)$ is:

(30) $$\cos\left(\beta \right)=\frac{1}{4}\sum_{i=1}^{c}\mu_{1}^{i}\mu_{2}^{i}=\sum_{i=1}^{c}\sqrt{\theta_{1}^{i}\theta_{2}^{i}}.$$

The Riemannian distance between these two points is the arc length on the sphere:

(31) $$d\left(\theta_{1},\theta_{2}\right)=2\arccos\sum_{i=1}^{c}\sqrt{\theta_{1}^{i}\theta_{2}^{i}}.$$

In the regularization term defined in Equation (24), we replace δ with the following upper bound:

(32) $$\delta =d\left(f\left(x\right),\Delta^{m}\setminus A_{x}\right)\leq d\left(f(x),O\right),$$

where $O=\frac{1}{c}\left(1,\ldots ,1\right)$ is the center of the simplex $\Delta^{m}$. Thus,

(33) $$\delta \leq 2\arccos\sum_{i=1}^{c}\sqrt{\frac{f^{i}\left(x\right)}{c}}.$$

## 4. Experiments

The regularization method introduced in Section 3 is evaluated on MNIST and CIFAR-10 datasets. Our method uses the loss function introduced in Equation (25).

### 4.1. Experiments on MNIST Dataset

#### 4.1.1. Experimental Setup

For the MNIST dataset, we implement a LeNet model with two convolutional layers of 32 and 64 channels, respectively, followed by one hidden layer with 128 neurons. The code is available here: https://github.com/lshigarrier/geometric_robustness.git (accessed on 1 December 2022). We train three models: one regularized model, one baseline unregularized model, and one model trained with adversarial training. All three models are trained with the Adam optimizer ($\beta_{1}=0.9$ and $\beta_{2}=0.999$) for 30 epochs, with a batch size of 64, and a learning rate of $10^{-3}$. For the regularization term, we use a budget of ϵ=5.6, which is chosen to contain the $l_{\infty }$ ball of radius 0.2. The adversarial training is conducted with 10 iterations of PGD with a budget $\epsilon_{adv}=0.2$ using $l_{\infty }$ norm. We found that $\lambda =10^{-6}$ yields the best performance in terms of robustness–accuracy trade-off; this value is small because we did not attempt to normalize the regularization term.

The models are trained on the 60,000 images of MNIST’s training set and then tested on 10,000 images of the test set. The baseline model achieves an accuracy of 98.9% (9893/10,000), the regularized model achieves an accuracy of 94.0% (9403/10,000), and the adversarially trained model achieves an accuracy of 98.8% (9883/10,000). Although the current implementation of the regularized model is almost six times slower to train than the baseline model, it may be possible to accelerate the training using, for example, the technique proposed by Shafahi et al. [15], or using another method to approximate the spectral norm of $\tilde{J}$. Even without relying on these acceleration techniques, the regularized model is still faster to train than the adversarially trained model.

#### 4.1.2. Robustness to Adversarial Attacks

**Figure 3:**
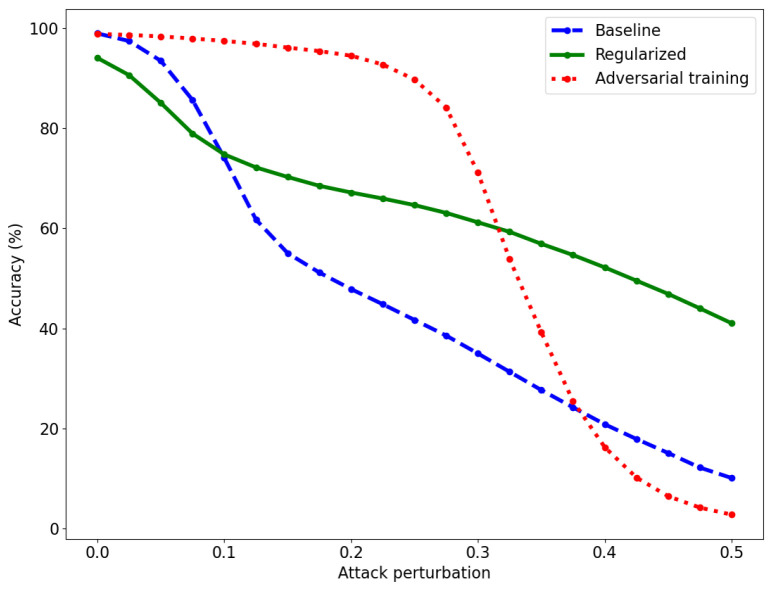
Accuracy of the baseline (dashed, blue), regularized (solid, green), and adversarially trained (dotted, red) models for various attack perturbations on the MNIST dataset. The perturbations are obtained with PGD using $l_{\infty }$ norm.

To measure the adversarial robustness of the models, we use the PGD attack with the $l_{\infty }$ norm, 40 iterations, and a step size of 0.01. The $l_{\infty }$ norm yields the hardest possible attack for our method, and corresponds more to the human notion of “indistinguishable images” than the $l_{2}$ norm. The attacks are performed on the test set, and only on images that are correctly classified by each model. The results are reported in Figure 3. The regularized model has a slightly lower accuracy than the baseline model for small perturbations, but the baseline model suffers a drop in accuracy above the attack level ϵ=0.1. Adversarial training achieves high accuracy for small- to medium-sized perturbations but the accuracy decreases sharply above ϵ=0.3. The regularized model remains robust even for large perturbations. The baseline model reaches 50% accuracy at ϵ=0.2 and the adversarially trained model at ϵ=0.325, while the regularized model reaches 50% accuracy at ϵ=0.4.

Table 1 provides more results against AutoAttack (AA) [7], which was designed to offer a more reliable evaluation of adversarial robustness. For a fair comparison, and in addition to a baseline model (BASE), we compare the partial isometry defense (ISO) with several other computationally efficient defenses: distillation (DIST) [8], Jacobian regularization (JAC) [9], which also relies on the Jacobian matrix of the network, and Fisher information regularization (FIR) [10], which also leverages information geometry. We also consider an adversarially trained (AT) model using PGD. ISO is the best defense that does not rely on adversarial training. In future work, ISO may be combined with AT to further boost performance. Note that ISO and JAC are more robust against $l_{2}$ attacks since they were designed to defend the model against such attacks. On the other hand, AT is more robust against $l_{\infty }$ attacks, because the adversarial training was conducted with the $l_{\infty }$ norm.

### 4.2. Experiments on CIFAR-10 Dataset

We consider a DenseNet121 model fine-tuned on CIFAR-10 using pre-trained weights for ImageNet. The code is available here: https://github.com/lshigarrier/iso_defense.git (accessed on 26 January 2023). As for the MNIST experiments, we compare the partial isometry defense with distillation (DIST), Jacobian regularization (JAC), and Fisher information regularization (FIR). Here, adversarial training (AT) relies on the fast gradient sign method (FGSM) attack [16]. All defenses are compared against PGD for various attack strengths. The results are presented in Table 2. The defenses are evaluated in a “gray-box” setting where the adversary can access the architecture and the data but not the weights. More precisely, the adversarial examples are crafted from the test set of CIFAR-10 using another unregularized DenseNet121 model. AT is the more robust method, but ISO achieves a robust accuracy 30% higher than the next best analogous method (FIR).

One of our goals is to provide alternatives to adversarial training (AT). Apart from high computational costs, AT suffers from several limitations: it only robustifies against the chosen attack at the chosen budget and it does not offer a robustness guarantee. For example, under Gaussian noise, AT accuracy decreases faster than baseline accuracy (i.e., no defense). Achieving high robustness accuracy against specific attacks on a specific benchmark is insufficient and misleading to measure the true robustness of the evaluated model. Our method offers a new point of view that can be extended to certified defense methods in future works.

## 5. Discussion and Related Work

In 2019, Zhao et al. [17] proposed to use the Fisher information metric in the setting of adversarial attacks. They used the eigenvector associated with the largest eigenvalue of the pullback of the FIM as an attack direction. Following their work, Shen et al. [10] suggested a defense mechanism by suppressing the largest eigenvalue of the FIM. They upper-bounded the largest eigenvalue by the trace of the FIM. As in our work, they added a regularization term to encourage the model to have smaller eigenvalues. Moreover, they showed that their approach is equivalent to label smoothing [18]. In our framework, their method consists of expanding the geodesic ball $\tilde{b}\left(x,\delta \right)$ as much as possible. However, their approach does not guarantee that the constraint imposed on the model will not harm the accuracy more than necessary. In our framework, matrix PJ (compared with δ/ϵ) informs the model on the precise restriction that must be imposed to achieve adversarial robustness in the $l_{2}$ ball of radius ϵ.

Cisse et al. [19] introduced another adversarial defense called Parseval networks. To achieve adversarial robustness, the authors aim to control the Lipschitz constant of each layer of the model to be close to unity. This is achieved by constraining the weight matrix of each layer to be a Parseval tight frame, which is another name for semi-orthogonal matrix. Since the Jacobian matrix of the entire model with respect to the input is almost the product of the weight matrices, the Parseval network defense is similar to our proposed defense, albeit with completely different rationales. This suggests that geometric reasoning could successfully supplement the line of work on Lipschitz constants of neural networks, such as in [20].

Following another line of work, Hoffman et al. [9] advanced a Jacobian regularization to improve adversarial robustness. Their regularization consists of using the Frobenius norm of the input–output Jacobian matrix. To avoid computing the true Frobenius norm, they relied on random projections, which are shown to be both efficient and accurate. This method is similar to the method of Shen et al. [10] in the sense that it will also increase the radius of the geodesic ball. However, the Jacobian regularization does not take into account the geometry of the output space (i.e., the Fisher information metric) and assumes that the probability simplex $\Delta^{m}$ is Euclidean.

Although this study focuses on $l_{2}$ norm robustness, it must be pointed out that there are other “distinguishability” measures that can be used to study adversarial robustness, including all other $l_{p}$ norms. In particular, the $l_{\infty }$ norm is often considered to be the most natural choice when working with images. However, the $l_{\infty }$ norm is not induced by any inner product and, hence, there is no Riemannian metric that induces the $l_{\infty }$ norm. However, given an $l_{\infty }$ budget $\epsilon_{\infty }$, we can choose an $l_{2}$ budget $\epsilon_{2}=\sqrt{d}\epsilon_{\infty }$, such that any attack in the $\epsilon_{\infty }$ budget will also respect the $\epsilon_{2}$ budget. When working on images, other dissimilarity measures are rotations, deformations, and color changes of the original image. Contrary to the $l_{2}$ or $l_{\infty }$ norms, these measures do not rely on a pixel-based coordinate system. However, it is possible to define unrestricted attacks based on these spatial dissimilarities, for example, in [21].

In this work, we derive the partial isometry regularization for a classification task. The method can be extended to regression tasks by considering the family of multivariate normal distributions as the output space. On the probability simplex $\Delta^{m}$, the FIM is a metric with constant positive curvature, while it has constant negative curvature on the manifold of multivariate normal distributions [22].

Finally, the precise quantification of the robustness condition presented in Equation (12) and Proposition 4 paves the way for the development of a certified defense [23] in this framework. By strongly enforcing Proposition 4 on a chosen proportion of the training set, it may be possible to maximize the accuracy under the constraint of a chosen robustness level, which offers another solution to the robustness–accuracy trade-off [24,25]. Certifiable defenses are a necessary step for the deployment of deep learning models in critical domains and missions, such as civil aviation, security, defense, and healthcare, where a certification may be required to ensure a sufficient level of trustworthiness.

## 6. Conclusions and Future Work

In this paper, we introduce an information geometric approach to the problem of adversarial robustness in machine learning models. The proposed defense consists of enforcing a partial isometry between the input space endowed with the Euclidean metric and the probability simplex endowed with the Fisher information metric. We subsequently derived a regularization term to achieve robustness during training. The proposed strategy is tested on the MNIST and CIFAR-10 datasets, and shows a considerable increase in robustness without harming the accuracy. Future works will evaluate the method on other benchmarks and real-world datasets. Several attack methods will also be considered in addition to PGD and AutoAttack. Although this work focuses on $l_{2}$ norm robustness, future work will consider other “distinguishability” measures.

Our work extends a recent, promising but understudied framework for adversarial robustness based on information geometric tools. The FIM has already been harnessed to develop attacks [17] and defenses [10,26] but a precise robustness analysis is yet to be proposed. Our work is a step toward the development of such an analysis, which might yield certified guarantees relying on these geometric tools. The study of adversarial robustness, which is non-local by definition and contrary to accuracy, should benefit greatly from a geometrical vision. However, the current literature on adversarial robustness is mainly concerned with the FIM and its spectrum (which are very local objects) without unfolding the full arsenal developed in information geometry. In our work, we demonstrate the usefulness of such an approach by developing a preliminary robustification method. Model robustification is a hard, unsolved yet vital problem to ensure the trustworthiness of deep learning tools in safety-critical applications. Our framework could be extended and applied to existing certification strategies, such as Lipschitz-based [27] or randomized smoothing [23], where statistical models naturally appear.

## Figures and Tables

**Table 1:** Clean and robust accuracy on MNIST against AA, averaged over 10 runs. The number in parentheses is the attack strength.

Defense	BASE	ISO	DIST	JAC	FIR	AT
Clean	99.01	96.51	98.81	98.95	98.84	98.98
AA-$l_{2}$ (0.15)	35.70	43.38	35.35	38.74	1.68	73.34
AA-$l_{\infty }$ (1.5)	10.38	22.15	9.63	13.30	0.03	95.43

**Table 2:** Clean and robust accuracy on CIFAR-10 against PGD. The number in parentheses is the attack strength.

Defense	BASE	ISO	DIST	JAC	FIR	AT
Clean	92.93	76.86	84.96	86.17	89.98	80.78
PGD (4/255)	2.49	40.17	7.54	8.56	9.74	68.82
PGD (8/255)	0.47	39.68	3.35	3.66	4.05	66.61

## Data Availability

Publicly available datasets were analyzed in this study. This data can be found here: http://yann.lecun.com/exdb/mnist/ (accessed on 1 December 2022) and https://www.cs.toronto.edu/~kriz/cifar.html (accessed on 26 January 2023).

## Appendix A. Proofs

**Proof of Proposition** **2** (17) ⇒ (16). Assume (17). Let $v\in {\bar{B}}_{x}\left(0,\epsilon \right)$. Thus, ${\bar{g}}_{x}\left(v,v\right)\leq \epsilon^{2}$. We have

(A1) $${\tilde{g}}_{x}\left(v,v\right)\leq \frac{\delta^{2}}{\epsilon^{2}}{\bar{g}}_{x}\left(v,v\right)\leq \frac{\delta^{2}}{\epsilon^{2}}\epsilon^{2}=\delta^{2}.$$

Thus, $v\in {\tilde{B}}_{x}\left(0,\delta \right)$. (16) ⇒ (17). Assume (16). Let $v\in T_{x}X$, v≠0. Define $w=\epsilon \;v/\sqrt{{\bar{g}}_{x}\left(v,v\right)}$. Then ${\bar{g}}_{x}\left(w,w\right)=\epsilon^{2}$. Thus, $w\in {\bar{B}}_{x}\left(0,\epsilon \right)$. Hence, $w\in {\tilde{B}}_{x}\left(0,\delta \right)$. Thus, ${\tilde{g}}_{x}\left(w,w\right)<\delta^{2}$. Finally, we have

(A2) $${\tilde{g}}_{x}\left(w,w\right)=\frac{\epsilon^{2}}{{\bar{g}}_{x}\left(v,v\right)}{\tilde{g}}_{x}\left(v,v\right)<\delta^{2}.$$

We obtain Equation (17) by multiplying by ${\bar{g}}_{x}\left(v,v\right)/\epsilon^{2}$. □

**Proof of Fact** **1.** We prove the third equality (the second equality is a well-known fact of linear algebra). Let u∈kerJ. Then, $J^{T}GJu=0$; thus, $u\in \ker(J^{T}GJ)$. Hence, ${\left(\ker(J^{T}GJ)\right)}^{⊥}\subseteq {\left(\ker(J)\right)}^{⊥}$. Let $v\in \ker J^{T}GJ$. Since *G* is symmetric positive-definite, the function $w\mapsto N\left(w\right)=\sqrt{w^{T}Gw}$ is a norm. We have $0=v^{T}J^{T}GJv=N{\left(Jv\right)}^{2}$. The positive-definiteness of the norm *N* implies Jv=0. Thus, v∈kerJ. Hence, ${\left(\ker(J)\right)}^{⊥}\subseteq {\left(\ker(J^{T}GJ)\right)}^{⊥}$. □

**Proof of Proposition** **3.** The implication (22) ⇒ (21) is immediate (by double inclusion). Now, assume (21) holds. Let v∈D. Define $w_{1}=\epsilon \;v/\sqrt{{\bar{g}}_{x}\left(v,v\right)}$ and $w_{2}=\epsilon \;v/\sqrt{{\tilde{g}}_{x}\left(v,v\right)}$. Then, with a similar argument as in the proof of Proposition 2, we can obtain Equation (22). Note that $w_{2}$ is well-defined because v∉ker(J). □

**Proof of Proposition** **4.** Let us first introduce the polar decomposition. Let *A* be a m×d matrix. Define the absolute value of *A* by $\left|A\right|={\left(A^{T}A\right)}^{\frac{1}{2}}$. Note that the square root of $A^{T}A$ is well-defined because it is a positive-semidefinite matrix Define the linear map u:rg(|A|)→rg(A) by u(|A|x)=Ax for any $x\in R^{d}$. Using the fact that |A| is symmetric, we have that ${∥Ax∥}^{2}=x^{T}A^{T}Ax={\left(A^{T}Ax\right)}^{T}x={\left(|A\right|}^{2}{x)}^{T}x=x^{T}{\left|A\right|}^{T}|A|x={∥|A|x∥}^{2}$; thus, *u* is an isometry (we can arbitrarily extend *u* on the entire $R^{d}$, e.g., by setting ker(u)=ker(|A|)). Let *U* be the matrix associated to *u* in the canonical basis. We now prove the main result. Let A=PJ. Using the polar decomposition, we have

(A3) PJ=U|PJ|,

where *U* is an isometry from $\mathrm{rg}\left(|PJ|\right)={\left(\ker|PJ|\right)}^{⊥}={\left(\ker\left(PJ\right)\right)}^{⊥}={\left(\ker\left(J\right)\right)}^{⊥}=D$ to $\mathrm{rg}\left(PJ\right)=R^{m}$ (using our assumption that rk(J)=m). Transposing this relation, we obtain

(A4) $$J^{T}P^{T}=\left|PJ\right|U^{T}.$$

Hence, by multiplying both relations, we have

(A5) $$PJJ^{T}P^{T}=U{\left|PJ\right|}^{2}U^{T}=UJ^{T}P^{T}PJU^{T}$$

Assume that (ii) holds, i.e., $PJJ^{T}P=I_{m}$. Then,

(A6) $$J^{T}GJ=J^{T}P^{T}PJ=U^{T}PJJ^{T}P^{T}U=U^{T}U.$$

Since *U* is an isometry from *D* to $R^{m}$, then $U^{T}U$ is the projection onto *D*, denoted as $\Pi_{D}$. Thus, we have $J^{T}GJ=\Pi_{D}$, which is (i). Now, assume that (i) holds, i.e., $J^{T}P^{T}PJ=\Pi_{D}$, where $\Pi_{D}$ is the projection onto *D*. We have

(A7) $$PJJ^{T}P^{T}=UJ^{T}P^{T}PJU^{T}=U\Pi_{D}U^{T}.$$

Since $\mathrm{rg}(U^{T})=D$, then $\Pi_{D}U^{T}=U^{T}$. Since *U* is an isometry from *D* to $R^{m}$, then $UU^{T}=I_{m}$. Thus, $PJJ^{T}P^{T}=I_{m}$ which is (ii). □

**Proof of Proposition** **5.** We need to show that $\mu^{*}\bar{g}=g$. Using the coordinates θ on $\Delta^{m}$ (Definition 1) and the standard coordinates on $R^{c}$, and writing $f\left(x\right)=\theta_{0}=\left(\theta_{0}^{1},\ldots ,\theta_{0}^{m}\right)$ we have

$$\begin{array}{cc} G_{ij} & =G_{\theta_{0},ij},\\ & =\sum_{\alpha =1}^{c}\sum_{\beta =1}^{c}\frac{\partial \mu^{\alpha }\left(\theta_{0}\right)}{\partial \theta^{i}}\frac{\partial \mu^{\beta }\left(\theta_{0}\right)}{\partial \theta^{j}}\delta_{\alpha \beta },\\ & =\sum_{\alpha =1}^{c}\frac{\partial \mu^{\alpha }\left(\theta_{0}\right)}{\partial \theta^{i}}\frac{\partial \mu^{\alpha }\left(\theta_{0}\right)}{\partial \theta^{j}}. \end{array}$$

For i=1,…,m and α=1,…,m we have

(A8) $$\frac{\partial \mu^{\alpha }\left(\theta_{0}\right)}{\partial \theta^{i}}=\frac{\delta_{i\alpha }}{\sqrt{\theta_{0}^{i}}},$$

and for α=c:

(A9) $$\frac{\partial \mu^{c}\left(\theta_{0}\right)}{\partial \theta^{i}}=-\frac{1}{\sqrt{\theta_{0}^{c}}},$$

with $\theta_{0}^{c}=\sqrt{1-\sum_{i=1}^{m}\theta_{0}^{i}}$. Thus,

(A10) $$G_{\theta ,ij}=\frac{\delta_{ij}}{\theta_{0}^{i}}+\frac{1}{\theta_{0}^{c}},$$

which is the FIM, as defined in Definition 2. □

**Proof of Proposition** **6.** For i=1,…,m, the inverse transformation of τ(μ) is

(A11) $$\mu^{i}\left(\tau \right)=\frac{2\tau^{i}}{1+{∥\tau /2∥}^{2}},$$

and

(A12) $$\mu^{c}\left(\tau \right)=2\frac{{∥\tau /2∥}^{2}-1}{{∥\tau /2∥}^{2}+1}.$$

The proofs of Equations (A11) and (A12) are provided below. Moreover, according to Proposition 5, the FIM in the coordinates $\left(\mu^{1},\ldots ,\mu^{m}\right)$ is the metric induced on $\mu (\Delta^{m})$ by the identity matrix (i.e., the Euclidean metric) of $R^{c}$. Hence, we have

$$\begin{array}{cc} G_{\tau ,ij} & =\sum_{\alpha =1}^{c}\sum_{\beta =1}^{c}\frac{\partial \mu^{\alpha }\left(\tau \right)}{\partial \tau^{i}}\frac{\partial \mu^{\beta }\left(\tau \right)}{\partial \tau^{j}}\delta_{\alpha \beta },\\ & =\sum_{\alpha =1}^{c}\frac{\partial \mu^{\alpha }\left(\tau \right)}{\partial \tau^{i}}\frac{\partial \mu^{\alpha }\left(\tau \right)}{\partial \tau^{j}}. \end{array}$$

For i=1,…,m and α=1,…,m, we have

(A13) $$\frac{\partial \mu^{\alpha }\left(\tau \right)}{\partial \tau^{i}}=\frac{2}{{1+∥\tau /2∥}^{2}}\left(\delta_{i\alpha }-\frac{\tau^{\alpha }\tau^{i}}{2\left({1+∥\tau /2∥}^{2}\right)}\right),$$

and for α=c:

(A14) $$\frac{\partial \mu^{c}\left(\tau \right)}{\partial \tau^{i}}=\frac{2\tau^{i}}{{\left({1+∥\tau /2∥}^{2}\right)}^{2}},$$

Thus,

$$\begin{array}{cc} G_{\tau ,ij}= & \frac{4}{{\left({1+∥\tau /2∥}^{2}\right)}^{2}}(\sum_{\alpha =1}^{m}\{\delta_{i\alpha }\delta_{j\alpha }-\frac{\delta_{i\alpha }\tau^{j}\tau^{\alpha }}{2\left({1+∥\tau /2∥}^{2}\right)}-\frac{\delta_{j\alpha }\tau^{i}\tau^{\alpha }}{2\left({1+∥\tau /2∥}^{2}\right)}+\frac{\tau^{i}\tau^{j}{\left(\tau^{\alpha }\right)}^{2}}{4{\left({1+∥\tau /2∥}^{2}\right)}^{2}}\}+\frac{\tau^{i}\tau^{j}}{{\left({1+∥\tau /2∥}^{2}\right)}^{2}}),\\ = & \frac{4}{{\left({1+∥\tau /2∥}^{2}\right)}^{2}}(\delta_{ij}-\frac{\tau^{i}\tau^{j}}{{1+∥\tau /2∥}^{2}}+\frac{\tau^{i}\tau^{j}{∥\tau /2∥}^{2}}{{\left({1+∥\tau /2∥}^{2}\right)}^{2}}+\frac{\tau^{i}\tau^{j}}{{\left({1+∥\tau /2∥}^{2}\right)}^{2}}),\\ = & \frac{4}{{\left({1+∥\tau /2∥}^{2}\right)}^{2}}\left(\delta_{ij}-\frac{\tau^{i}\tau^{j}}{{1+∥\tau /2∥}^{2}}+\frac{\tau^{i}\tau^{j}}{{1+∥\tau /2∥}^{2}}\right),\\ = & \frac{4}{{\left({1+∥\tau /2∥}^{2}\right)}^{2}}\delta_{ij}. \end{array}$$

□

**Proof of Equations** **(A11)** **and** **(A12).** We have $\tau^{i}\left(\mu \right)=\lambda \mu^{i}$ with $\lambda =2/\left(2-\mu^{c}\right)$. Let us express $\mu^{c}$ as a function of τ. We have

(A15) $${∥\tau ∥}^{2}=\sum_{i=1}^{m}{\left(\tau^{i}\right)}^{2}=\lambda^{2}{∥\mu ∥}^{2}.$$

Since μ belongs to the sphere of radius 2, we have ${∥\mu ∥}^{2}+{\left(\mu^{c}\right)}^{2}=4$. Thus,

(A16) $${∥\tau ∥}^{2}=\lambda^{2}\left(4-{\left(\mu^{c}\right)}^{2}\right)=4\frac{4-{\left(\mu^{c}\right)}^{2}}{{\left(2-\mu^{c}\right)}^{2}}=4\frac{2+\mu^{c}}{2-\mu^{c}}.$$

Isolating $\mu^{c}$, we obtain

(A17) $$\mu^{c}\left(\tau \right)=\frac{{2∥\tau ∥}^{2}-8}{{∥\tau ∥}^{2}+4}=2\frac{{∥\tau /2∥}^{2}-1}{{∥\tau /2∥}^{2}+1}.$$

Now, we can replace $\mu^{c}$ with the expression of λ. We obtain $\lambda =\left({1+∥\tau /2∥}^{2}\right)/2$; thus,

(A18) $$\mu^{i}\left(\tau \right)=\frac{\tau^{i}}{\lambda }=\frac{2\tau^{i}}{{1+∥\tau /2∥}^{2}}$$

□

**Proof of Proposition** **7.** We have

(A19) $$\tau^{i}\left(\theta \right)=2\sqrt{\theta^{i}}/\left(1-\sqrt{\theta^{c}}\right).$$

Thus,

$$\begin{array}{cc} {∥\frac{\tau (\theta )}{2}∥}^{2} & =\sum_{i=1}^{m}\frac{\tau^{i}{\left(\theta \right)}^{2}}{4}=\frac{\sum_{i=1}^{m}\theta^{i}}{{\left(1-\sqrt{\theta^{c}}\right)}^{2}}=\frac{1-\theta^{c}}{{\left(1-\sqrt{\theta^{c}}\right)}^{2}}=\frac{1+\sqrt{\theta^{c}}}{1-\sqrt{\theta^{c}}}. \end{array}$$

Hence, for any i=1,…,m:

(A20) $$\frac{2}{{1+∥\tau \left(\theta \right)/2∥}^{2}}=1-\sqrt{\theta^{c}}=\frac{2\sqrt{\theta^{i}}}{\tau^{i}\left(\theta \right)}.$$

Now, we compute $\tilde{J}$. Let *i* and *j* in {1,…,m}:

(A21) $$\begin{array}{cc} \frac{\partial \tau^{i}\left(\theta \right)}{\partial \theta^{j}} & =\frac{\delta_{ij}}{\sqrt{\theta^{i}}\left(1-\sqrt{\theta^{c}}\right)}-\frac{\sqrt{\theta^{i}}}{\sqrt{\theta^{c}}{\left(1-\sqrt{\theta^{c}}\right)}^{2}}, \end{array}$$

(A22) $$\begin{array}{cc} \; & =\frac{\tau^{i}\left(\theta \right)}{2}\left(\frac{\delta_{ij}}{\theta^{i}}-\frac{\tau^{i}\left(\theta \right)}{2\sqrt{\theta^{i}\theta^{c}}}\right). \end{array}$$

Replacing Equations (A20) and (A22) with Equation (28) yields the result. □
